# Antibacterial activity and mechanism of sanguinarine against *Staphylococcus aureus* by interfering with the permeability of the cell wall and membrane and inducing bacterial ROS production

**DOI:** 10.3389/fvets.2023.1121082

**Published:** 2023-03-30

**Authors:** Yeqing Gu, Jun Dong, Jing Li, Qianmin Luo, Xianlan Dong, Guowen Tang, Jiaxiang Zhang, Xuan Du, Qiqi Pu, Lin He, Kaiwei Zhao, Diangang Han, Jige Xin

**Affiliations:** ^1^College of Veterinary Medicine, Yunnan Agricultural University, Kunming, China; ^2^Animal Quarantine Laboratory, Technology Center of Kunming Customs, Kunming, China; ^3^College of Plant Protection, Yunnan Agricultural University, Kunming, China

**Keywords:** sanguinarine, sanguinarine chloride hydrate, *Staphylococcus aureus*, cell wall and membrane damage, oxidative damage

## Abstract

*Staphylococcus aureus* (*SA*) is representative of gram-positive bacteria. Sanguinarine chloride hydrate (SGCH) is the hydrochloride form of sanguinarine (SG), one of the main extracts of Macleaya cordata (M. cordata). There are few reports on its antibacterial mechanism against *SA*. Therefore, in this study, we investigated the *in vitro* antibacterial activity and mechanism of SGCH against *SA*. The inhibitory zone, minimum inhibitory concentration (MIC), and minimum bactericidal concentration (MBC) were measured, and the bactericidal activity curve was plotted. In addition, the micromorphology, alkaline phosphatase (AKP) activity, Na^+^K^+^, Ca^2+^Mg^2+^-adenosine triphosphate (ATP) activity, intracellular reactive oxygen species (ROS), and fluorescein diacetate (FDA) were observed and detected. The results showed that the inhibitory zone of SGCH against *SA* was judged as medium-sensitive; the MIC and MBC were 128 and 256 μg/mL, respectively; in the bactericidal activity curve, SGCH with 8 × MIC could completely kill *SA* within 24 h. SGCH was able to interfere with the integrity and permeability of the *SA* cell wall and membrane, as confirmed by the scanning electron microscopy (SEM) images, the increase in extracellular AKP and Na^+^ K^+^, Ca^2+^ Mg^2+^-ATP activities as well as the fluorescein diacetate (FDA) staining experiment results. Moreover, a high concentration of SGCH could induce *SA* to produce large amounts of ROS. In summary, these findings revealed that SGCH has a preferable antibacterial effect on *SA*, providing an experimental and theoretical basis for using SG as an antibiotic substitute in animal husbandry and for the clinical control and treatment of diseases caused by *SA*.

## 1. Introduction

Since the discovery and invention of antimicrobial drugs, these compounds have played an important role in the treatment of human diseases and are widely used in agriculture and animal husbandry (1). However, with the abuse of antibiotics, the problem of drug resistance has become increasingly serious. In 2018, the World Health Organization (WHO) held a conference and identified antimicrobial resistance as “the greatest and most urgent global risk” (2). The long-term and heavy use of antimicrobial drugs can not only cause drug residues in livestock and poultry, resulting in the mechanisms of antimicrobial resistance (AMR) and multi-drug resistance in animal organisms, but also may produce variant strains (3). If we eat too many livestock and poultry products that use these drugs, as the drugs are accumulated in the livestock and poultry, and then transmitted to humans through the food chain and water environment. Humans may also easily develop drug resistance, and when humans are infected with viruses or bacteria, these antibacterial drugs may not work in humans (4–6). Strictly controlling the violation and abuse of antimicrobial drugs will not only prevent food safety issues, but also protect human health. Therefore, it is of great significance to find and develop alternatives to antibiotics to ensure the safety and stability of animal production and to achieve sustainable animal husbandry (7).

Macleaya cordata (M. cordata) is a traditional medicinal herb in Macleaya (Papaveraceae) with strong ecological adaptability and abundant resources in China (8). Sanguinarine (SG) is one of the main extracts of M. cordata, which primarily accumulates in the leaves, accounting for approximately 61% of the total alkaloid content. It belongs to one of the benzophenanthridine alkaloids, with the molecular formula of C_20_H_14_NO_4_ and the molecular weight of 332.33 (9). SG has excellent insecticidal properties and unique anti-tumor effects. It also has the advantages of being antibacterial (10) and anti-inflammatory (11), improving the growth performance of young animals (12), enhancing immunity (13), and promoting appetite (14). Sanguinarine chloride hydrate (SGCH) is the hydrochloride form of sanguinarine (SG), with the molecular formula of C_20_H_14_ClNO_4_ and the molecular weight of 367.78, the character is orange needle-like crystals, and the melting point is 281–285°C. It has the advantages of fast absorption, rapid distribution, fast metabolism, low bioavailability, and low body residue, and it has excellent effects on various livestock and poultry breeding efforts (15). Khin et al. (16) evaluated the synergy, additivity, and antagonism effects of alkaloids in Macleaya cordata extract (MCE). Their tests showed that SG and chelerythrine had obvious antibacterial effects on methicillin-resistant *S. aureus* AH1263 and multiply-resistant *S. aureus* IA116. Xia et al. (17) found that MCE inhibited the bacterial amplification and suppressed the expression of proinflammatory cytokines *in vivo*, as well as promoted the suppression of immune-related gene expression to prevent the excessive inflammation and improve the integrity of damaged spleens. Other studies (18) have shown that SG also has a great antibacterial effect through intraperitoneal injection of carp with *Aeromonas hydrophila*. Zhang et al. (19) investigated the antibacterial activity and mechanism of SG against *Providencia rettgeri* and showed that SG could exhibit inhibitory effects on its biofilm formation and disrupt the integrity and permeability of the cell membrane. Moreover, Quinteros et al. (20) showed that SG could effectively inhibit the growth of *Campylobacter hepaticus* (spotty liver disease) and significantly reduce the proinflammatory cytokines in the blood of infected hens. Hu et al. (21) found that SG had obvious inhibitory and inactivating effects on *Candida albicans in vivo* and *in vitro*. On the same side, Wang et al., (22) showed that SG could reduce blood eosinophils and serum inflammatory factors, relieve subclinical mastitis, improve milk quality, and facilitate the recovery of acute tissue damage and health without affecting the production performance and rumen function of dairy cows.

*Staphylococcus aureus* (*SA*) is representative of gram-positive bacteria, and is a facultative anaerobic, non-spore-forming and highly reproductive bacterium (23). It can survive in dry and hot conditions and can also reproduce in hypersaline environments (24). *SA* can cause not only localized pyogenic infections but also arthritis and endocarditis. The toxins released by *SA* can cause non-specific systemic inflammation, leading to uncontrollable sepsis (25). In livestock and poultry breeding, *SA* is one of the main pathogens causing bacterial infectious diseases, including mastitis, vaginitis and endometritis of dairy cows and sheep (26). Poultry infected with *SA* may even have acute septicaemia symptoms, resulting in mass mortality (27). *SA* has obvious resistance to common antibiotic treatment, but the preferred method for treating bacterial diseases is still antibiotic therapy. Consequently, there is an urgent need to find and develop novel drugs for treating *SA* infections that are unlikely to develop antibiotic resistance but are also easy to degrade. Therefore, we investigated the antibacterial activity and antibacterial mechanism *in vitro* of SGCH against *SA* with the hope of providing theoretical support for the application of SG in animal husbandry and clinical treatment.

## 2. Materials and methods

### 2.1. Source and preparation of strains

The *Staphylococcus aureus* [*SA*, CMCC (B) 26003] used in this test was provided by the Animal Quarantine Laboratory of Kunming Customs Technology Center (Kunming, China). The frozen tube containing magnetic beads of the strain was removed from the refrigerator at −80°C. Then, one bead was added to a sterile centrifuge tube containing 5 mL of nutrient broth (NB) and incubated overnight at 37°C with shaking at 150 rpm. The next day the centrifuge tube containing the bacterial solution was removed, and the bacterial solution was diluted to 0.5 Malcolm turbidity with sterile deionized water (DI water), at which time the bacterial concentration was approximately 1.0 × 10^8^ CFU/mL. Lastly, the solution was diluted 1:100 to a concentration of approximately 1.0 × 10^6^ CFU/mL as the test solution.

### 2.2. Source and preparation of test drug

Sanguinarine chloride hydrate (SGCH) was purchased from McLean Biochemical Technology Co. (S817437, ≥ 98% (HPLC), Shanghai, China), due to the poor water solubility of SGCH, 0.3% dimethyl sulfoxide (DMSO, Solarbio, D8371, Beijing, China) was used as the cosolvent in this test, and 10 mg/mL was prepared as the stock solution; chlortetracycline hydrochloride (CTC) is a positive control, which was purchased from Solabao Technology Co. (C9100, Beijing, China), also prepared with 0.3% DMSO into 5 mg/mL as the stock solution. Both were stored at 4°C.

### 2.3. Determination of the inhibitory zone diameter of SGCH against *SA*

First, 100 μL of *SA* was added to a sterile culture dish, then 15 mL of agar medium was cooled to 50°C and poured, shaken rapidly and gently until well mixed, the agar was allowed to cool and solidify, and 6 mm diameter holes were punched on the agar plate with a sterile puncher. The holes were spaced more than 2.5 cm apart. Then, 0.3% DMSO, 5 mg/mL CTC, and 250, 500, and 1,000 μg/mL SGCH were added clockwise at 15 μL per well respectively. Lastly, the dishes were inverted and incubated at 37°C for 16–18 h, and the diameter of the inhibitory zone was measured and recorded using vernier calipers. When measuring the inhibitory zone, a uniform and completely sterile growing zone of inhibition should be selected for the measurement.

This zone was calculated as follows:


$$\begin{array}{l} \text{Inhibitory zone diameter }=\text{ total diameter }-\text{ pore diameter}. \end{array}$$

### 2.4. MIC and MBC

The MIC and MBC were determined in accordance with the methods of Heuser et al. (28). In brief, using the microdouble diffusion method, wells judged to be clarified by the naked eye were recorded as MIC; using the agar diffusion method, the concentration at which there was no colony growth at all was recorded as the MBC.

### 2.5. The bactericidal activity curve of SGCH against *SA*

The bactericidal activity curves were plotted according to Sihotang et al. (29). When the suspensions (0, 8, 4, 2, and 1 × MIC) were cultured for 0, 1, 2, 3, 6, 12, and 24 h, 10 μL was taken for tenfold dilution five times, and 20 μL of each dilution was placed in a sterile culture dish, and mixed rapidly with 15 mL agar medium, and the mixtures were then inverted and incubated at 37°C for 16–18 h. The total number of a colony at a concentration of 20–300 was taken for counting, and the CFU average was calculated to derive the original colony concentrations in the different centrifuge tubes at different time points. The time point was used as the horizontal coordinate, and the usual logarithm of the number of bacteria (CFU) contained per mL was used as the vertical coordinate to plot the bactericidal activity curve using GraphPad Prism 8.0 software.

The concentration was calculated as follows:


$$\begin{array}{l} \text{The original colony concentration}\\ \;=\text{the average number of colonies from three}\\ \text{ replicates at the same dilution }\\ \;\times \text{ the dilution multiple }\times \;10\text{ CFU}/\text{mL}. \end{array}$$

### 2.6. Observations of SGCH on the morphology of *SA* cells by SEM

First, SGCH was added to the *SA* suspensions at the logarithmic stage to give a final concentrations of 0 × MIC, 8 × MIC, 4 × MIC, and 2 × MIC. The centrifuge tubes were incubated at 37°C with shaking at 150 rpm, and 10 mL was sampled when the tubes were incubated until 0, 6, 12, and 24 h. Then, the samples were centrifuged at 5,000 rpm for 10 min, and the supernatant was removed. Next, 2 mL of 2.5% glutaraldehyde was added to the precipitate, which was vortexed for 2 min to distribute the bacterial solution in the fixative solution evenly. The tube was wrapped with aluminum paper and then left in the dark for 12 h at 4°C in the refrigerator for full fixation so that the cell morphology could no longer change. During this time, an ethanol gradient eluent was configured (30%, 50%, 70%, 80%, 90%, 95%, and 100%). The fixed solution was removed and then centrifuged and the supernatant was removed. The resulting precipitate was added to 1 mL of PBS to make the suspensions visually relatively turbid and then washed with PBS three times. The solutions were then dehydrated step by step with 1 mL of 30% ethanol, vortexed and shaken for 3 min, allowed to stand for 5 min, and centrifuged at 5,000 rpm for 10 min. The supernatant was discarded, and the other volume fractions of ethanol solution were added in turn. Until the last 1 mL of 100% ethanol was added, the mixture was vortexed and shaken for 3 min, allowed to stand for 5 min, and ultimately pipetted onto the front and back of a small coverslip. After the coverslip had dried, the sample was obtained. The conductive adhesive was glued to the sample stage. Then, the coverslips were carefully glued to the conductive adhesive and coated with a high vacuum coater, which was then applied to the SEM (Hitachi SEM Flex1000 II, Japan), and the sample could be observed after the critical drying of the system.

### 2.7. Determination of SGCH on AKP activity of *SA*

AKP activities were determined according to the method of Liu et al. (30), and supernatants from 0 × MIC, 8 × MIC, 4 × MIC, and 2 × MIC that had been incubated for 0, 6, 12, and 24 h were treated with an alkaline phosphatase (AKP) activity kit (Nanjing Jiancheng Bioengineering Institute, A059-2-2, Nanjing, China). The absorbance values were measured at OD520 nm on a full wavelength microplate reader (Thermo Fisher Multiskan Sky, Shanghai, China). The changes in AKP activities were used to reflect the effects of SGCH on the bacterial cell wall.

### 2.8. Determination of SGCH on Na^+^ K^+^, Ca^2+^ Mg^2+^-ATP activity of *SA*

Supernatants from 0 × MIC, 8 × MIC, 4 × MIC, and 2 × MIC incubated for 0, 6, 12, and 24 h were treated with a Na^+^ K^+^, Ca^2+^ Mg^2+^-ATP activity kit (Nanjing Jiancheng, A070-5-2, Nanjing, China), and the absorbance values were measured at OD636 nm on a full wavelength microplate reader. The changes in Na^+^ K^+^and Ca^2+^ Mg^2+^-ATP activities were used to reflect the effects of SGCH on ion transport across *SA* cell membranes.

### 2.9. Fluorescein diacetate (FDA) staining experiment

SGCH was added to the SA suspensions at the logarithmic stage to give final concentrations of 0 × MIC, 8 × MIC, 4 × MIC, and 2 × MIC. The suspensions were incubated at 37°C with shaking at 150 rpm, and 1 mL was sampled at 0, 6, 12, and 24 h and then centrifuged at 5,000 rpm for 5 min and the supernatant was discarded. The bacteria were resuspended in 1 mL of PBS, and 42 μL FDA (Macklin, F809625, 50 mg/mL, Shanghai, China) dissolved in DMSO was added. After standing at room temperature for 20 min, the cells were resuspended and washed twice with PBS. Lastly, the supernatants were centrifuged, and the bacteria were resuspended in 1 mL of PBS. The fluorescence intensity was measured on a multifunctional microplate reader at an excitation wavelength of 297 nm and an emission wavelength of 527 nm.

### 2.10. Experiments on the intracellular ROS contents of *SA*

#### 2.10.1. Determination of the fluorescence intensity of ROS

ROS Measurement was performed according to Tang et al. (31). In brief, DCFH-DA (Beyotime Biotechnology, S0033S, Shanghai, China) was incubated with the bacteria for 20 min at 37°C and washed with PBS 3 times. PBS, 1024, 512, and 256 μg/mL of SGCH corresponding to 0 × MIC, 8 × MIC, 4 × MIC, and 2 × MIC were then added to the centrifuge tubes, and allowed to stand in the room temperature, and were incubated until 10, 20, and 30 min. Then 1 mL was sampled. The fluorescence intensity was measured on a multifunctional microplate reader (Thermo Fisher Varioskan LUX, Shanghai, China) at an excitation wavelength of 488 nm and an emission wavelength of 525 nm, and the fluorescence spectra were plotted.

#### 2.10.2. Observations of the green fluorescence of ROS

First, 3–5 μL of 0 × MIC and 8 × MIC suspensions treated for 30 min were added to glass slides, followed by 50 μL of antifade mounting medium (Beyotime Biotechnology, P0126, Shanghai, China). The coverslips were used to carefully cover the slide and were placed upside down under CLEM (Leica SP5 II, Germany) and observed by oil immersion at an excitation wavelength of 488 nm and an emission wavelength of 525 nm.

### 2.11. Statistical analysis

The test data were statistically processed with Excel, and the results were expressed as the mean values ± standard deviations (SD). GraphPad Prism 8.0 software was used to perform one-way ANOVA and multiple comparisons. All the experiments were performed independently at least three times. ^*^indicates a significant difference at the 0.05 level, ^**^indicates a significant difference at the 0.01 level and ^***^indicates a significant difference at the 0.001 level.

## 3. Results

### 3.1. Inhibitory zone diameter of SGCH at different concentrations against *SA*

SGCH (250, 500, 1,000 μg/mL) was screened for antibacterial activity and its activity was compared with chlortetracycline hydrochloride (CTC), which was also a positive control, and dimethylsulfoxide (DMSO) served as a negative control. The inhibitory ability increased in concert with increasing SGCH concentration (Table 1). SGCH (1,000 μg/mL) has higher antibacterial activity, with an average inhibitory zone of 15.67 mm, and it is judged to be medium-sensitive, but the activity of three SGCH concentrations was lower than that of 5 mg/mL CTC. The results suggested that different SGCH concentrations all had the ability to inhibit the growth of *SA*.

**Table 1:** Inhibitory zone diameter of SGCH at different concentrations against *SA*.

**Treatment**	**Inhibitory zone diameter (mm)**
0.3% DMSO	0 ± 0
5 mg/mL CTC	29.42 ± 0.8
250 μg/mL SGCH	11.5 ± 0.45
500 μg/mL SGCH	13.75 ± 0.69
1,000 μg/mL SGCH	15.67 ± 0.41

If the diameter of inhibitory zone is ≤7 mm, it is judged as no inhibitory effect. If it is >7 mm but <10 mm, it is judged to be low-sensitive; >10 mm but <20 mm, it is judged to be medium-sensitive and >20 mm is judged to be high-sensitive.

### 3.2. MIC and MBC

MIC and MBC were measured using broth microdilution and agar dilution methods. The MIC and MBC of SGCH against *SA* are 128 and 256 μg/mL, respectively; its MBC is twice that of MIC. In the following experiments, 256, 512, and 1024 μg/mL were used as 2 × MIC, 4 × MIC, and 8 × MIC of SGCH, respectively; PBS or 0.3% DMSO was used as 0 × MIC.

### 3.3. The bactericidal activity curve of SGCH against *SA*

**Figure 1:**
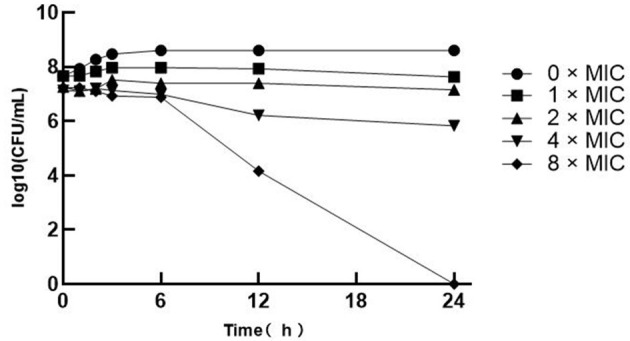
The Bactericidal Activity Curve of SGCH against *SA*. The curve from top to bottom in the figure are 0 × MIC, 1 × MIC, 2 × MIC, 4 × MIC and 8 × MIC, respectively.

The bacterial suspensions were blended into 0, 1, 2, 4, and 8 × MIC treatments and incubated for 0, 1, 2, 3, 6, 12, and 24 h. The number of colonies was counted and analyzed using the bactericidal activity curve (Figure 1). The *SA* at 0 × MIC showed a rapid increase and started to enter the logarithmic growth phase from 3 to 5 h and converged to the slow growth phase after 6 h, so the total bacterial colonies tended to be stable. However, there was little difference between the bactericidal effects of 1 × MIC and 2 × MIC, and colonies were still generated after 24 h. However, with the increase in the MIC of multiple SGCH concentrations, the bactericidal ability against *SA* was also improved. Both the 4 × MIC and 8 × MIC groups started to decrease significantly after 6 h, and there were no colonies in the 8 × MIC group after 24 h of culture, indicating that SGCH at 8 × MIC could kill *SA* within 24 h. The results showed that SGCH had a concentration dependence against *SA*.

### 3.4. Effects of SGCH on the morphology of *SA* cell

**Figure 2:**
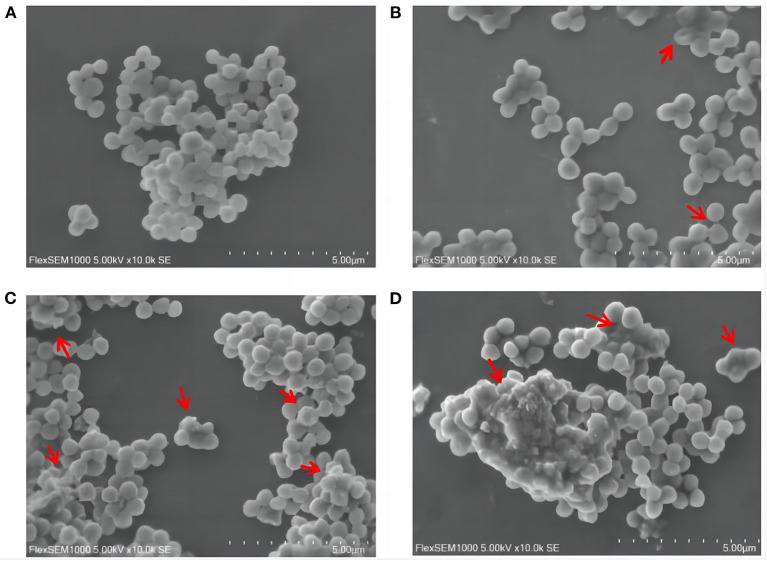
Effects of SGCH on the morphology of *SA* cells. **(A)** 0 × MIC group treated with 0.3% DMSO for 24 h. **(B)** 2 × MIC group treated with SGCH for 24 h. **(C)** 4 × MIC group treated with SGCH for 24 h. **(D)** 8 × MIC group treated with SGCH for 24 h. The arrows in the figure indicate *SA* cells had obvious damage.

Scanning electron microscopy (SEM) images further presented significant changes in cell morphology, and the integrity or damage to the cell structure could be observed. *SA* cells in the control group were rounded and plump with smooth surfaces (Figure 2A). On the contrary, the group treated with SGCH exhibited irregular shapes and rough surfaces (Figure 2B); some cells showed plenty of surface collapse, some were severely disrupted (Figure 2C), and most of the *SA* cells adhered to each other and agglomerated (Figure 2D).

### 3.5. Effects of SGCH on AKP activity of *SA*

**Figure 3:**
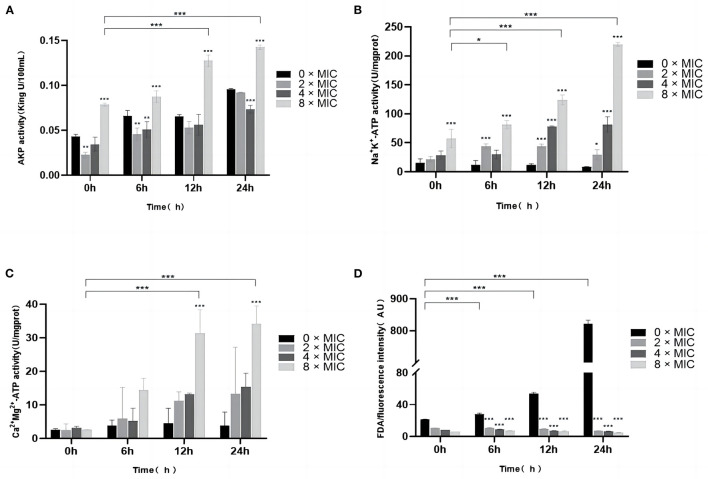
Effects of SGCH on AKP and Na^+^ K^+^, Ca^2+^ Mg^2+^-ATP Activities and FDA of *SA*. **(A)** Impact of SGCH on AKP Activity of *SA*. **(B)** Determination of *SA* Na^+^ K^+^-ATP activity by SGCH. **(C)** Determination of *SA* Ca^2+^ Mg^2+^-ATP activity by SGCH. **(D)** Fluorescein diacetate (FDA) staining experiment. *p ≤ *0.05*, ** *p* ≤ *0.01*, ***p ≤ *0.001*.

AKP is located between the cell walls and the cell membranes and can be used as an indicator of cell wall integrity. As shown in Figure 3A, all the concentrations of bacterial suspensions showed different degrees of increase in AKP activity after 6, 12, and 24 h, and the AKP activity at 8 × MIC, 4 × MIC, and 2 × MIC increased with the SGCH concentration and incubation time. At 0 h, 0 × MIC and 8 × MIC showed higher AKP activity. The 0 × MIC group might be caused by the turnover of old and new cells in *SA* itself, and the 8 × MIC group might have shown that result because the cell wall of *SA* was immediately disrupted as soon as the *SA* cells were exposed to a high concentration of SGCH, and AKP leaked from the cell, so both maintained higher AKP activity at the beginning. At 6 h, as the AKP activity of each concentration increased, there was a significant rise at 8 × MIC (*P* < *0.05*); and at 12 and 24 h, there was even a highly significant rise at 8 × MIC (*P* < *0.01*), which figured that a high concentration of SGCH could effectively disrupt the cell wall of *SA*, causing leakage of intracellular AKP and thus increasing extracellular AKP activity.

### 3.6. Effects of SGCH on Na^+^ K^+^, Ca^2+^ Mg^2+^-ATP activity of *SA*

ATP is a protein enzyme of biofilms and can be used as an indicator of cell membrane integrity. As shown in Figures 3B, C, in the control group, 0 × MIC maintained a relatively low level of ATP activity over all time periods. However, when bacterial suspensions were incubated for 24 h, the Na^+^ K^+^, Ca^2+^ Mg^2+^-ATP activities at 8 × MIC were highly significantly increased (*P* < 0.01). The results indicated that high SGCH concentrations could effectively disrupt the integrity of *SA* cell membranes and increase the permeability of Na^+^, K^+^and Ca^2+^, Mg^2+^, resulting in an increase in extracellular ATP activity in a concentration-dependent manner.

### 3.7. Fluorescein diacetate (FDA) staining experiment

FDA can produce yellow-green fluorescein, and when the cell membrane is damaged, the intracellular fluorescence intensity will dramatically decrease. As shown in Figure 3D, the fluorescence intensity of 0 × MIC was extremely significantly higher than that of 8 × MIC, 4 × MIC and 2 × MIC at 12 and 24 h (*P* < 0.01). The results showed that SGCH at different concentrations all significantly disrupted the cell membrane of *SA*, causing the loss of fluorescein from *SA* and thus reducing the fluorescence intensity of the FDA.

### 3.8. Effects of SGCH on the intracellular ROS content of *SA*

#### 3.8.1. Differences in the fluorescence intensity of ROS

**Figure 4:**
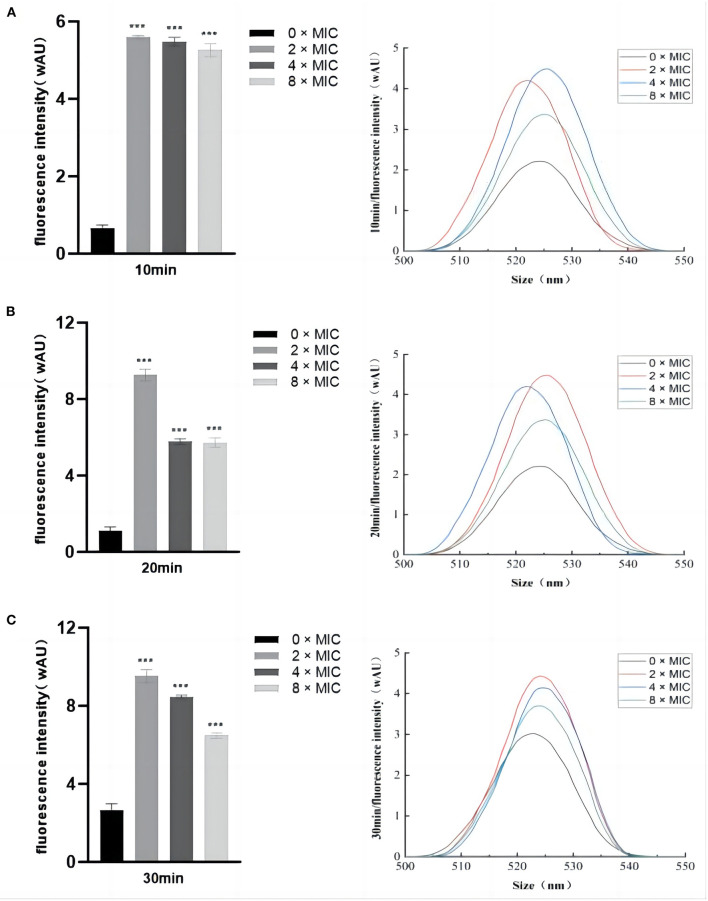
Differences in the fluorescence intensity of ROS. **(A)** Determination of *SA* intracellular ROS levels after SGCH treatment for 10 min; **(B)** Determination of *SA* intracellular ROS levels after SGCH treatment for 20 min; **(C)** Determination of *SA* intracellular ROS levels after SGCH treatment for 30 min. ***p < 0.001.

ROS act as important signaling molecules that reflects the state of cellular activity. The intracellular ROS levels after SGCH treatment with *SA* for 10 min, 20 min, and 30 min were measured fluorescence intensity. In the control group, the ROS content at 0 × MIC increased slowly with the increasing culture time (Figure 4). At 10, 20, and 30 min, the ROS contents at 8 × MIC, 4 × MIC, and 2 × MIC were highly significantly increased inversely (*P* < *0.01*). The most obvious increase in ROS content was observed for 2 × MIC, followed by 4 × MIC and 8 × MIC, which may have occurred because 2 × MIC was the most acceptable concentration. In addition, the plotted fluorescence spectroscopy was consistent with the fluorescence intensity data. The results showed that different concentrations of SGCH could induce *SA* to produce a large amount of ROS, thus causing bacterial oxidative damage.

#### 3.8.2. Differences in the green fluorescence of ROS

**Figure 5:**
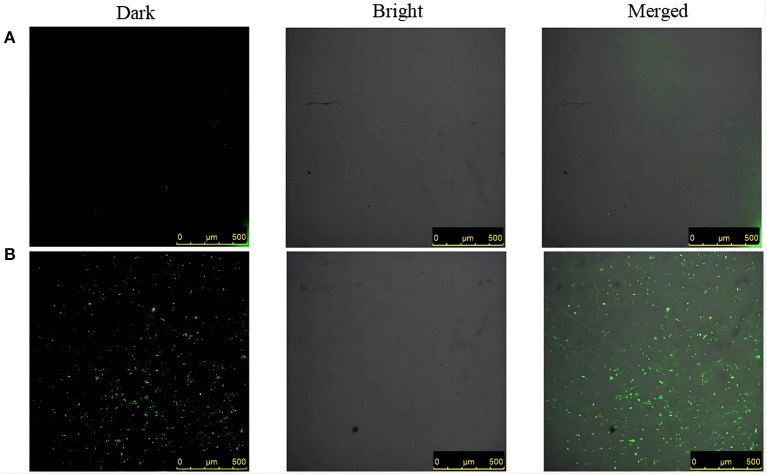
Differences in the green fluorescence of ROS. **(A)** 0 × MIC group treated with 0.3% DMSO for 30 min; **(B)** 8 × MIC group treated with SGCH for 30 min. From left to right are dark field, light field and merged field.

The intracellular ROS level after SGCH treatment of *SA* for 30 min was observed by confocal laser scanning microscope (CLEM). The green fluorescence represents DCFH-DA entering viable bacteria that release ROS during staining. At 30 min, in the control group, there was very little green fluorescence at 0 × MIC (Figure 5A). On the contrary, there was a great deal of green fluorescence at 8 × MIC, indicating that many bacteria produced a lot of ROS (Figure 5B). The results indicated that a high SGCH concentration could induce *SA* to produce large amounts of ROS, thus causing bacterial oxidative damage.

## 4. Discussion

With the intensive and large-scale development of animal husbandry in China, the incidence of bacterial diseases is also increasing. *SA* is one of the major pathogens causing bacterial infectious diseases. As early as 1999, Navarro studied the antibacterial effect of Bocconia arborea and found that dihydrochelerythrine and dihydrosanguinarine had the ability to inhibit the growth of gram-positive bacteria, with a stronger inhibitory effect on *Diplococcus pneumoniae, SA*, and *Bacillus subtilis* than berberine (13). Liang et al. (32) and Miao et al. (33) both found that SG compounds showed significant inhibition on *SA* and *Escherichia coli*. In this study, the *in vitro* antibacterial activity results showed that SGCH had a good inhibitory effect on *SA*, and SGCH (1,000 μg/mL) had a higher antibacterial activity, which were consistent with the results of these studies.

Many plant extracts play an antibacterial role by destroying the cell microstructure. Ren et al. (34) observed the effect of aureusidin on the *SA* micromorphology, and the results showed that the *SA* cells in the control group had a complete structure, but the group treated by aureusidin was partially dissolved and the cell surface became rough. Our results of SEM images showed that *SA* in the control group was round and full with a smooth surface. *SA* treated with SGCH showed collapse, surface dissolution, mutual adhesion, and leakage of cell sap, indicating that SGCH could obviously destroy the micromorphology and microstructure of SA cells.

The bacterial cell wall has the functions of protecting bacteria, transporting substances and participating in the pathogenic process. The damage of cell wall will affect its growth and the ability to resist the external environment. AKP is located between the cell wall and the cell membrane, which can be used as an indicator to detect the integrity of cell wall (35). In this study, the 8 × MIC group could induce the leakage of intracellular AKP, thus increasing the activity of extracellular AKP, which indicated that a high concentration of SGCH could effectively disrupt the cell wall of *SA*. He et al. (36) investigated the *in vitro* antibacterial mechanism of chelerythrine against *SA* and found that the extracellular AKP activity of bacteria in the experimental group was significantly increased, which was consistent with the results of this study.

The damage of cell membrane will cause the leakage of cytoplasm, which will seriously affect the metabolism of the bacterium. ATP exists on the tissue and organelle membranes and is a protease on the biofilm. It plays an important role in material transport, energy conversion, and information transmission. The determination of ATP activity values can be used as an indicator of the integrity of the cell membrane (37). In this experiment, the high SGCH concentration increased the permeability of Na^+^, K^+^and Ca^2+^, Mg^2+^, thus increasing the extracellular Na^+^ K^+^, Ca^2+^ Mg^2+^-ATP activities in a concentration-dependent manner, indicating that the high SGCH concentration can effectively destroy the integrity of the *SA* cell membrane. Tao et al. (38) measured the intracellular Ca^2+^ Mg^2+^-ATP activity of bacteria, which was significantly decreased in the experimental group, and the trend was consistent with that of this study.

After FDA is hydrolyzed by non-specific lipase in cells, it will produce fluorescein that can emit yellow-green fluorescence, and fluorescein will be detected at excitation and emission wavelengths of 297 nm and 527 nm, respectively. When the cell membrane is complete, fluorescein is present in the cell, and high-intensity fluorescence can be detected. When the cell membrane is disrupted, fluorescein will flow out of the cell quickly, and the intracellular fluorescence intensity will be greatly decreased. The permeability of the cell membrane can be reflected by the FDA fluorescence intensity values (39). The results of the FDA staining experiment in this study showed that different concentrations of SGCH all caused the loss of fluorescein from the intracellular bacteria, thus decreasing the fluorescence intensity of FDA, which indicated that SGCH at different concentrations could significantly damage the permeability of the *SA* cell membrane.

As an important signaling molecule, ROS can reflect the cellular activity state. Although cells can continuously produce low levels of ROS to maintain a normal cellular life activity, when the amount of intracellular ROS exceeds the capacity of the antioxidant mechanism, excessive oxidative stress will lead to irreversible damage to intracellular macromolecules (31). Tang et al. (31) explored the *in vitro* antibacterial mechanism of biogenic tellurium nanoparticles and precursor tellurite against *Escherichia coli*, and the measurement of ROS levels were found to be significantly higher in the experimental group. In this study, the fluorescence intensity, fluorescence spectroscopy, and CLEM results all reflected that different concentrations of SGCH could induce *SA* to produce a large amount of ROS, thus causing bacterial oxidative damage, which were consistent with this study.

*SA* is a representative pathogen causing a variety of diseases in humans and animals. It is sensitive to many antibiotics but also prone to drug resistance. In the context of global antibacterial restriction, there is an urgent need for safe and efficient alternative products in animal production, the development of new sterilization methods and the reduction of the use of antibiotics, so as to effectively control and prevent *SA* infections. Natural plant extracts are ideal alternatives to antibiotics in animal production because of their natural, multifunctional, low toxicity, high safety, and non-resistant characteristics (8). SG is the main active ingredient of M. cordata plants, and it has the advantages of fast absorption, rapid distribution, fast metabolism, low bioavailability, and low body residue in the organism, and will have excellent effects in various types of livestock and poultry breeding. Our results revealed that SGCH has a preferable antibacterial effect on *SA* so that SGCH could destroy the cellular structure of *SA*, interfere with the permeability of the *SA* cell wall and membrane, and induce oxidative damage in *SA*; thus, SGCH has the potential to be exploited as an antibiotic substitution in animal husbandry and for the clinical control and treatment of diseases caused by *SA*.

## 5. Conclusion

In conclusion, SGCH has good antibacterial activity against *SA*; Moreover, SGCH exerts its antibacterial mechanism by destroying the cellular structure, interfering with the permeability and integrity of the cell walls and membranes, and inducing *SA* oxidative damage. SGCH may thus be a potential antimicrobial agent for control and treatment of *SA* infections in the future research.

## Data availability statement

The original contributions presented in the study are included in the article/Supplementary material, further inquiries can be directed to the corresponding authors.

## Author contributions

Conceptualization: JX and YG. Methodology: YG and DH. Validation: JX and JD. Formal analysis: YG and JL. Investigation: QL. Resources: XDo. Data curation: GT and JZ. Writing—original draft preparation: YG. Writing—review and editing, project administration, and funding acquisition: DH and JX. Visualization: XDu and QP. Supervision: LH and KZ. All authors contributed to the article and approved the submitted version.
