# Supplementary figures and images for: Antibacterial activity and mechanism of sanguinarine against Staphylococcus aureus by interfering with the permeability of the cell wall and membrane and inducing bacterial ROS production
Source: Front Vet Sci. 2023 Mar 30;10:1121082. doi: 10.3389/fvets.2023.1121082 (PMC10101331; doi:10.3389/fvets.2023.1121082)

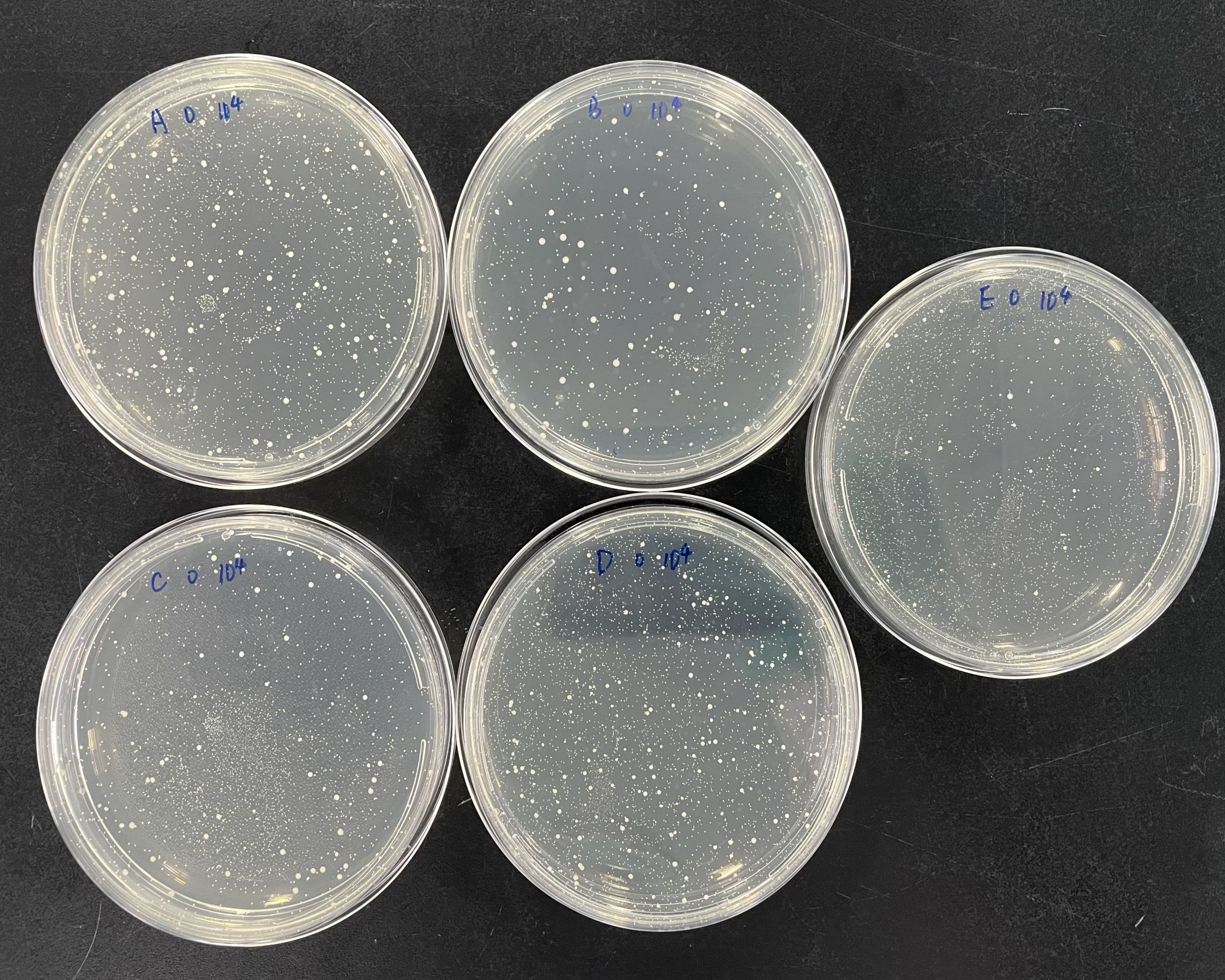

Supplement: Supplementary file 1 [file Data_Sheet_1.ZIP › Frontiers_raw data for Figure 1 and Table1-Gu Yeqing/Figure 1/0 × MIC.jpg]

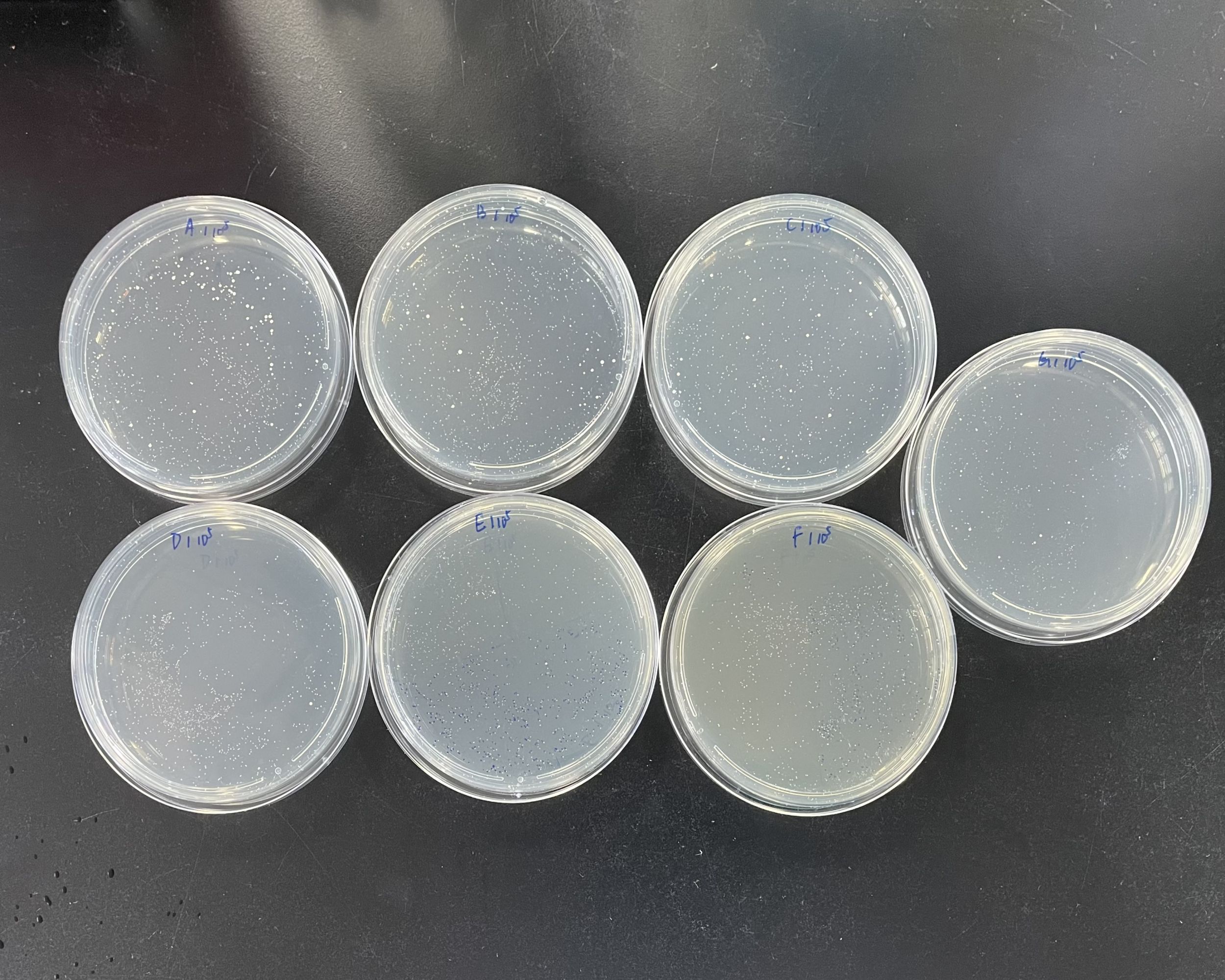

Supplement: Supplementary file 1 [file Data_Sheet_1.ZIP › Frontiers_raw data for Figure 1 and Table1-Gu Yeqing/Figure 1/1 × MIC.jpg]

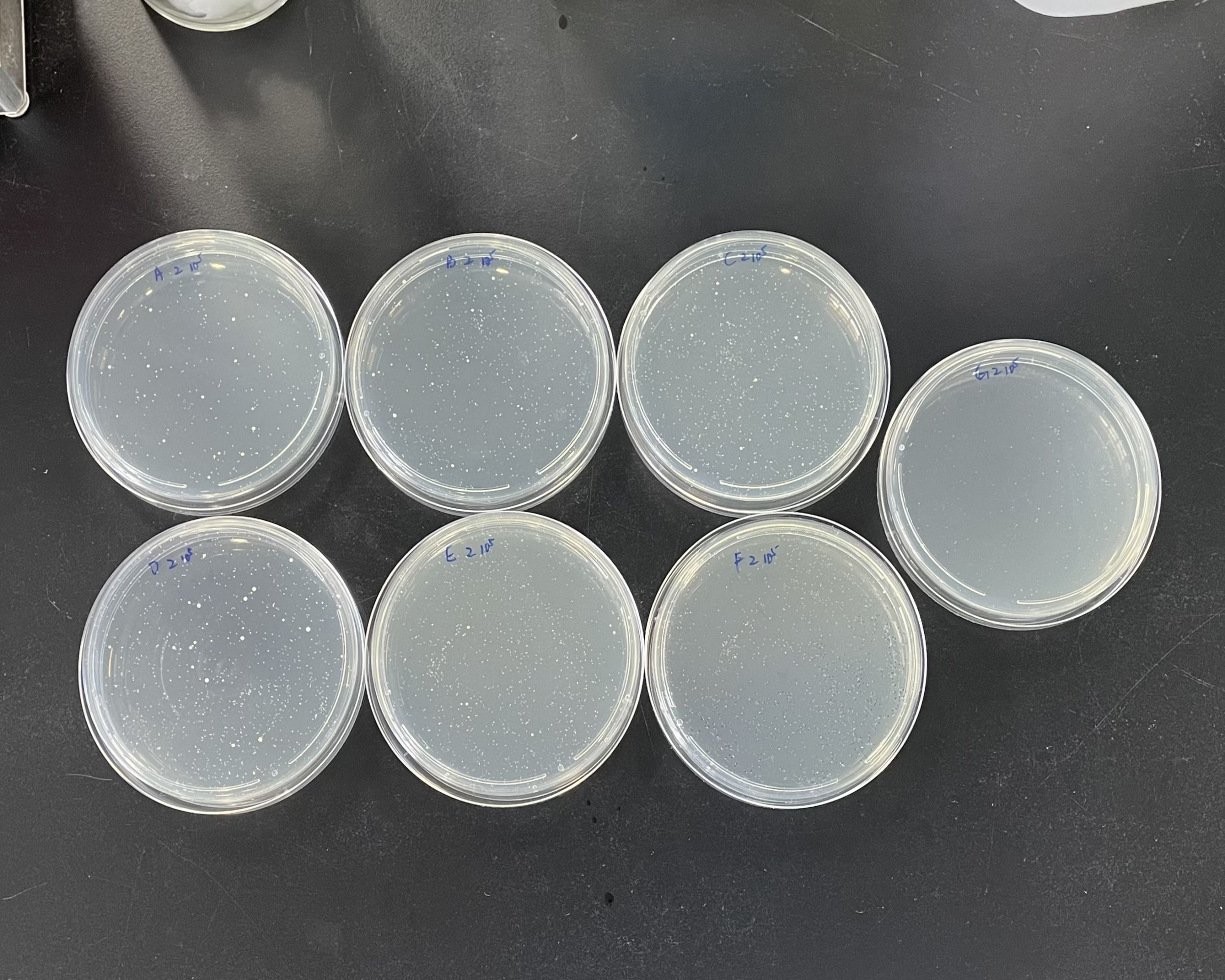

Supplement: Supplementary file 1 [file Data_Sheet_1.ZIP › Frontiers_raw data for Figure 1 and Table1-Gu Yeqing/Figure 1/2 × MIC.jpg]

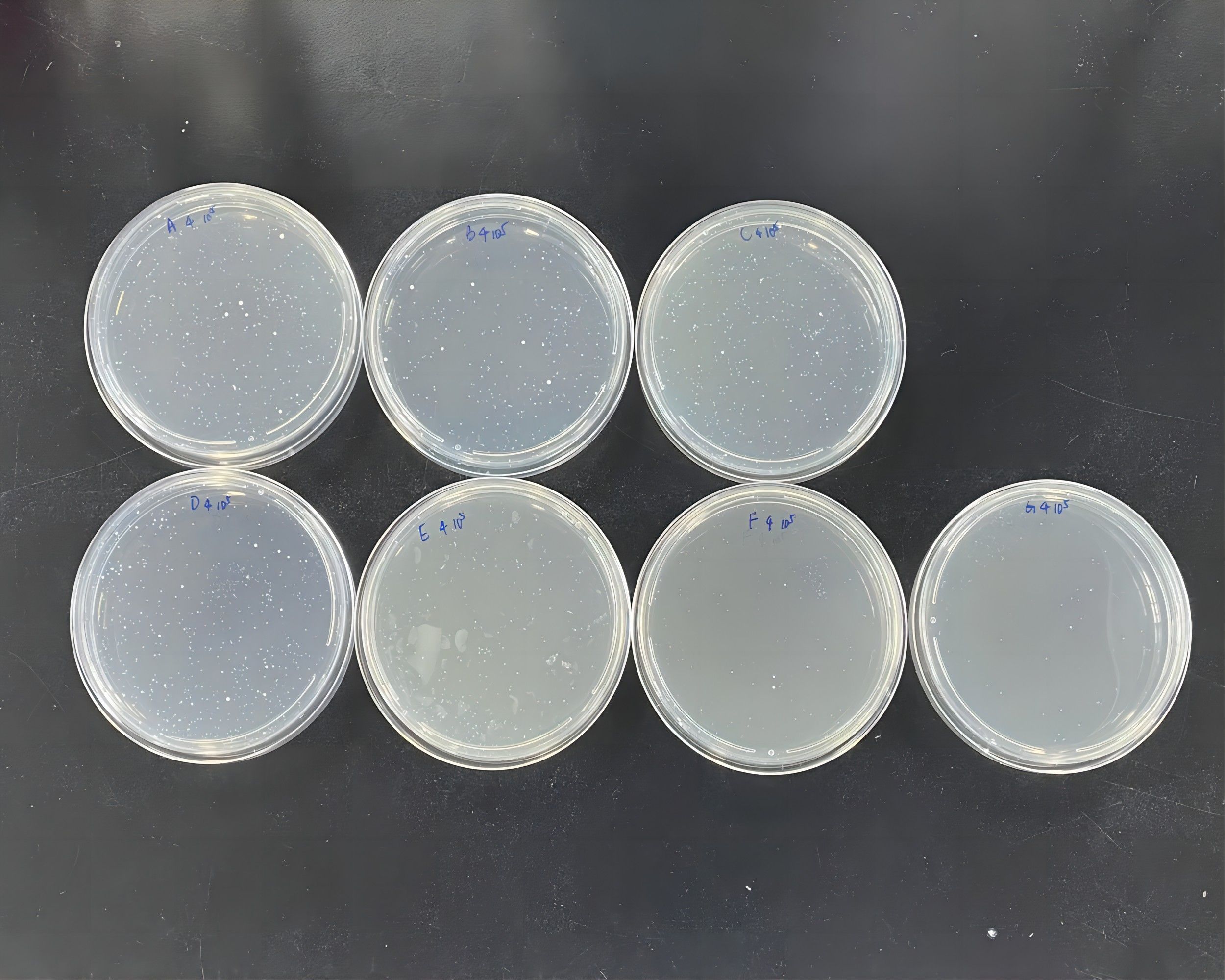

Supplement: Supplementary file 1 [file Data_Sheet_1.ZIP › Frontiers_raw data for Figure 1 and Table1-Gu Yeqing/Figure 1/4 × MIC.jpg]

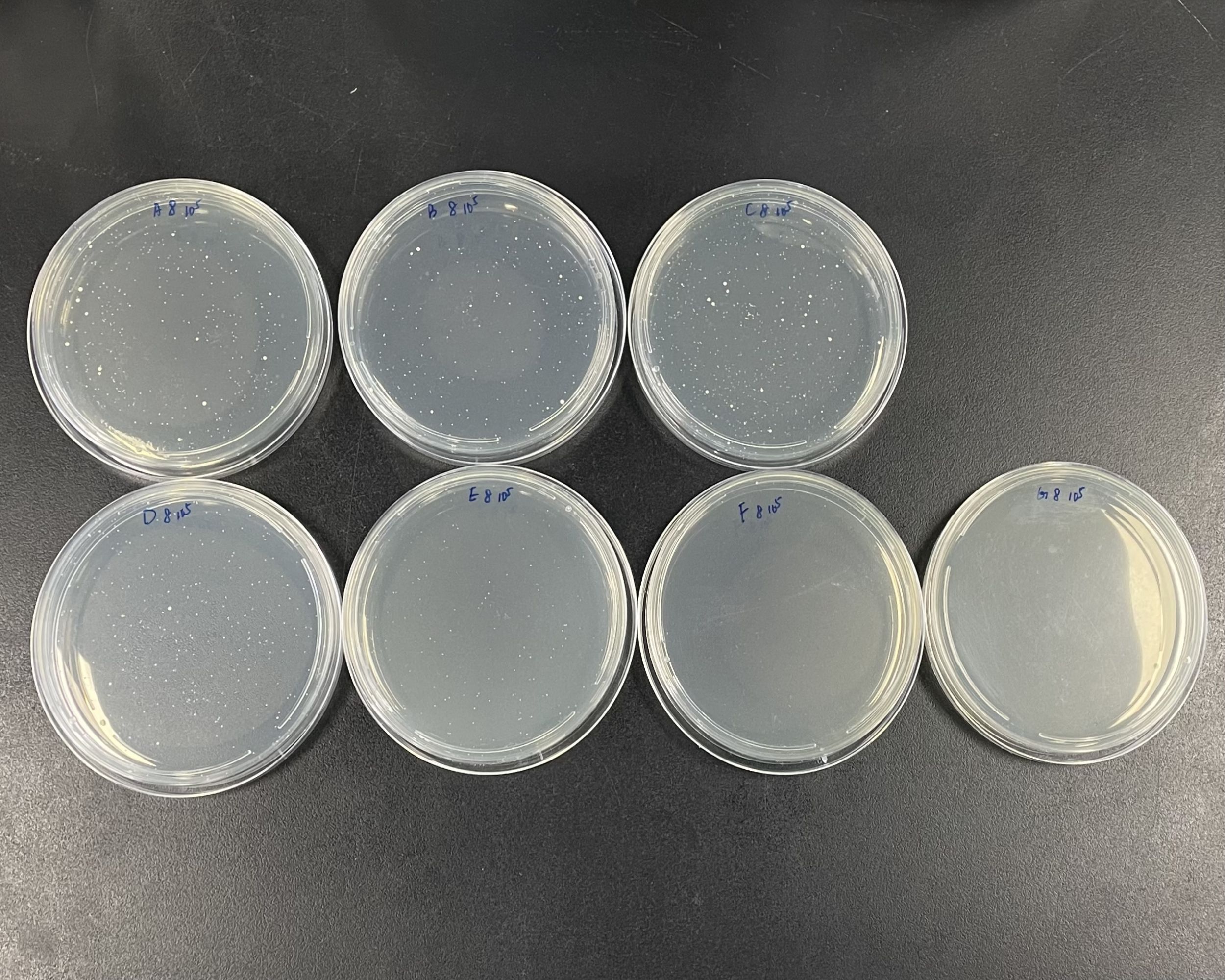

Supplement: Supplementary file 1 [file Data_Sheet_1.ZIP › Frontiers_raw data for Figure 1 and Table1-Gu Yeqing/Figure 1/8 × MIC.jpg]

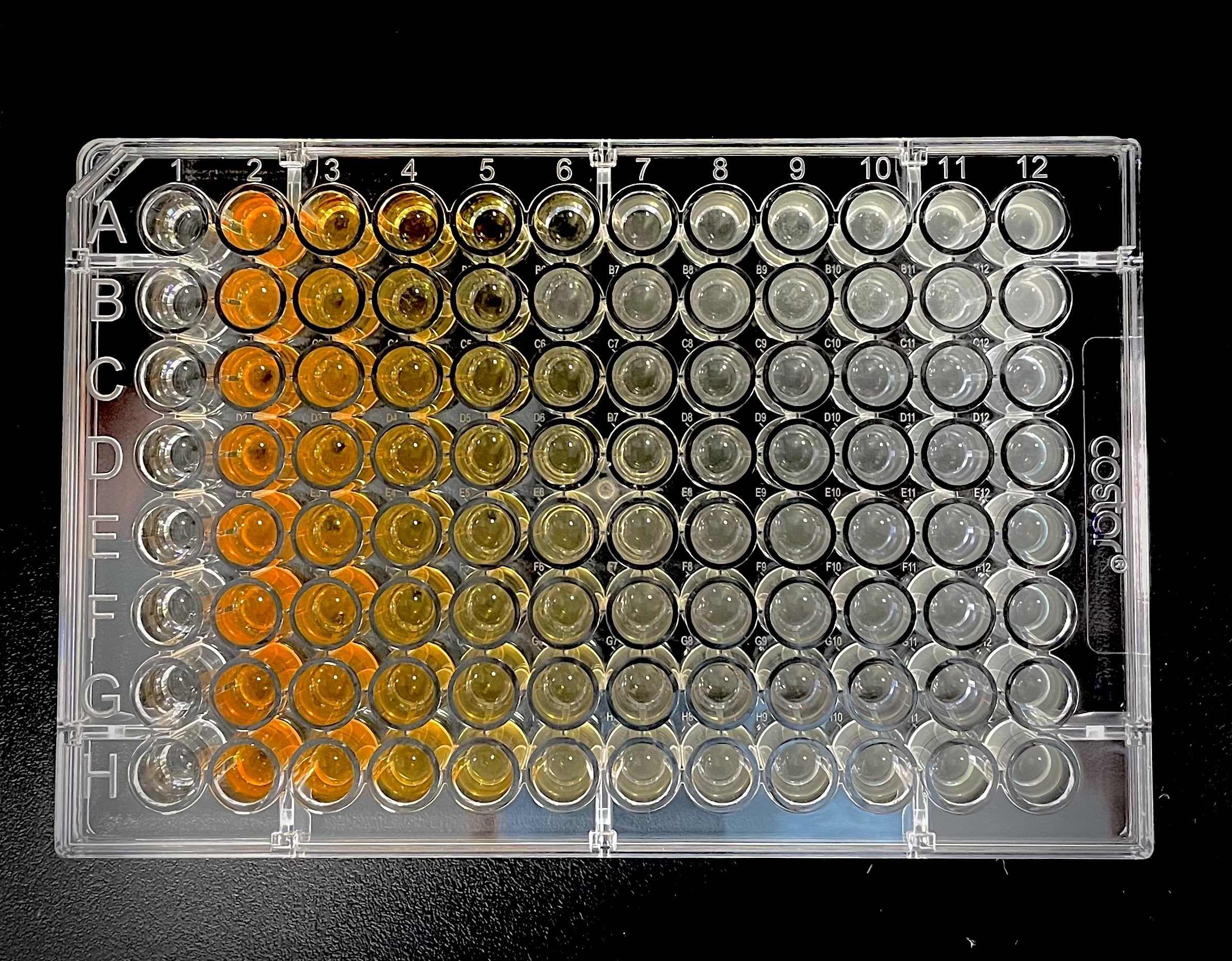


128 μg/mL


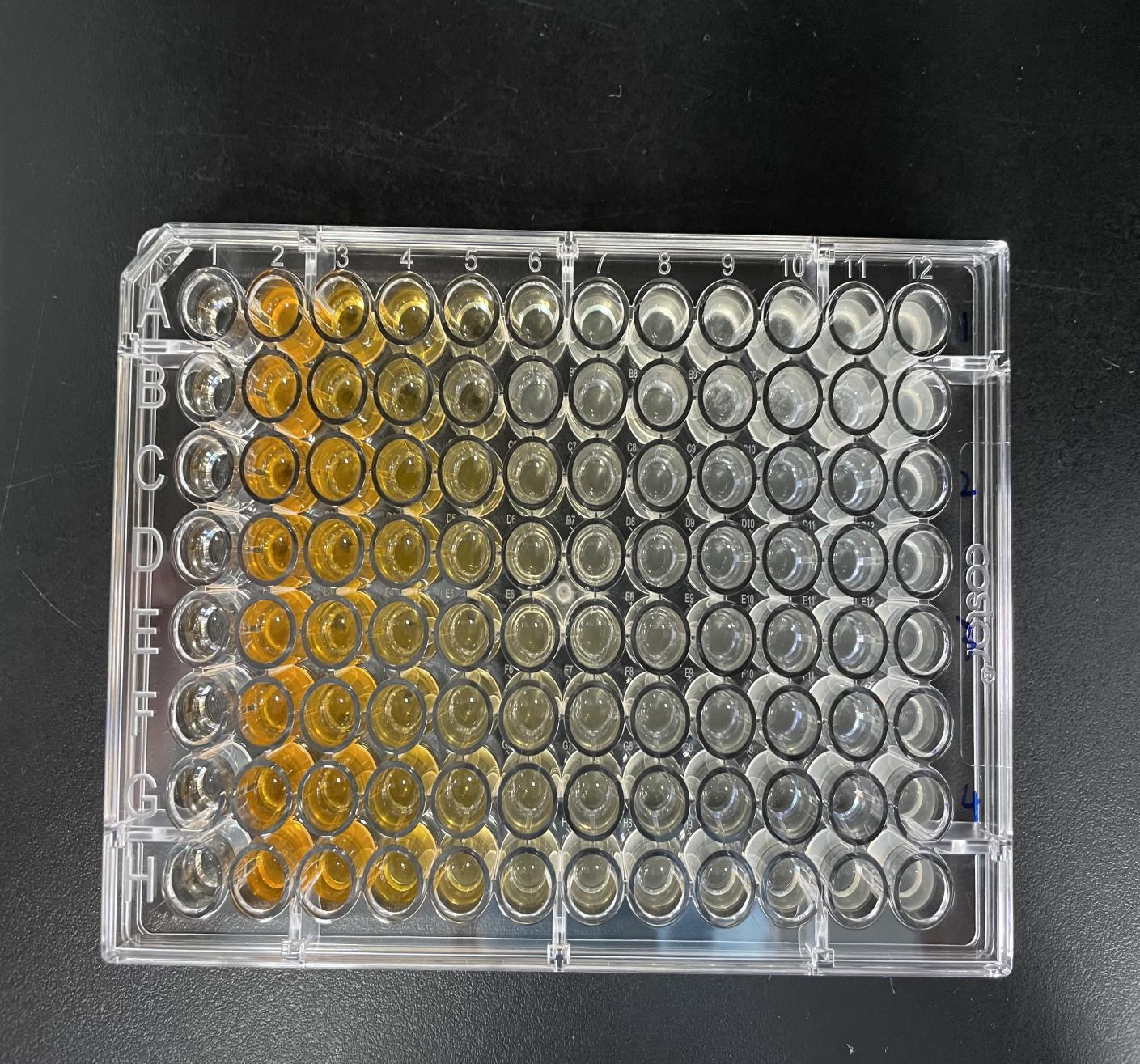


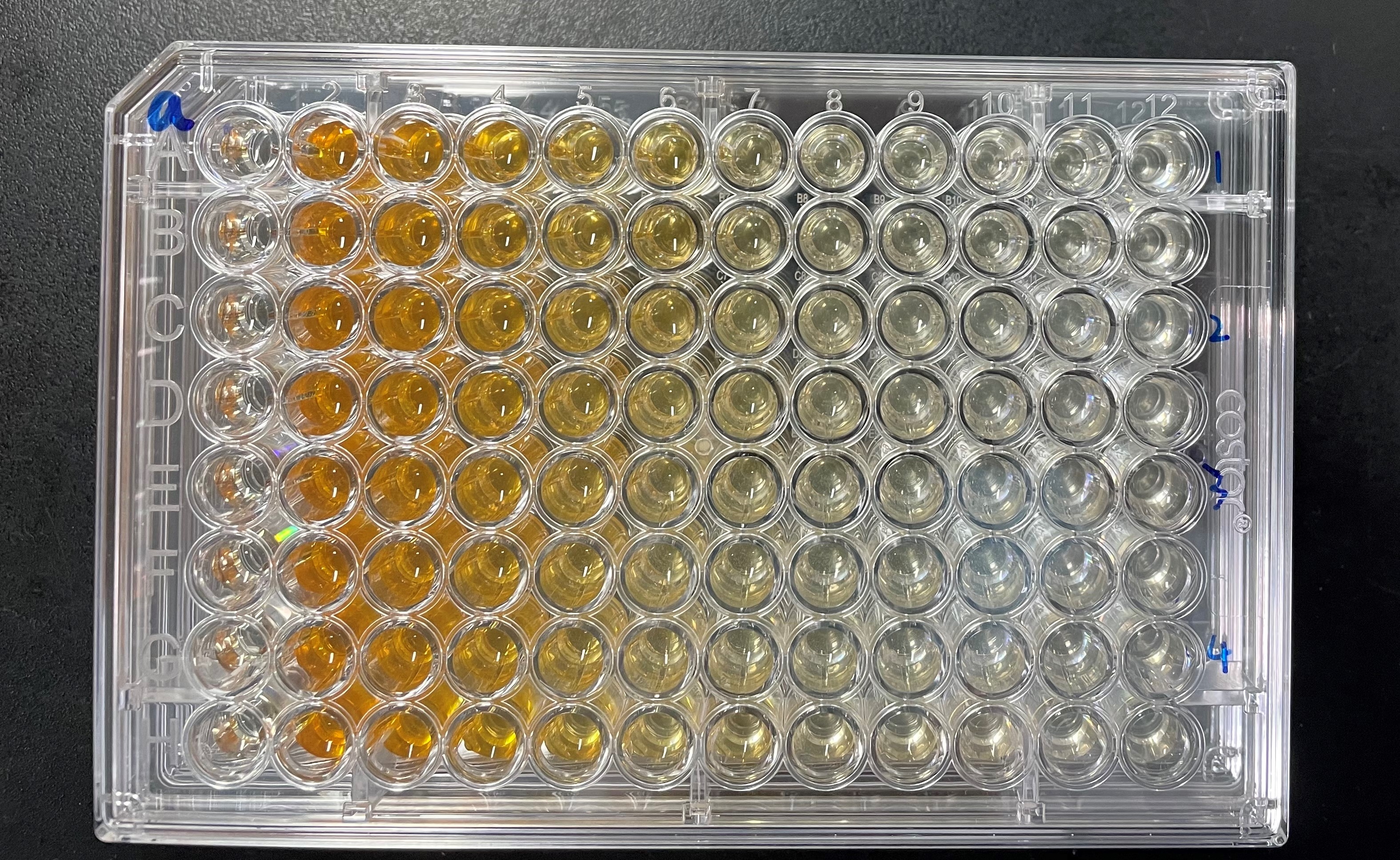

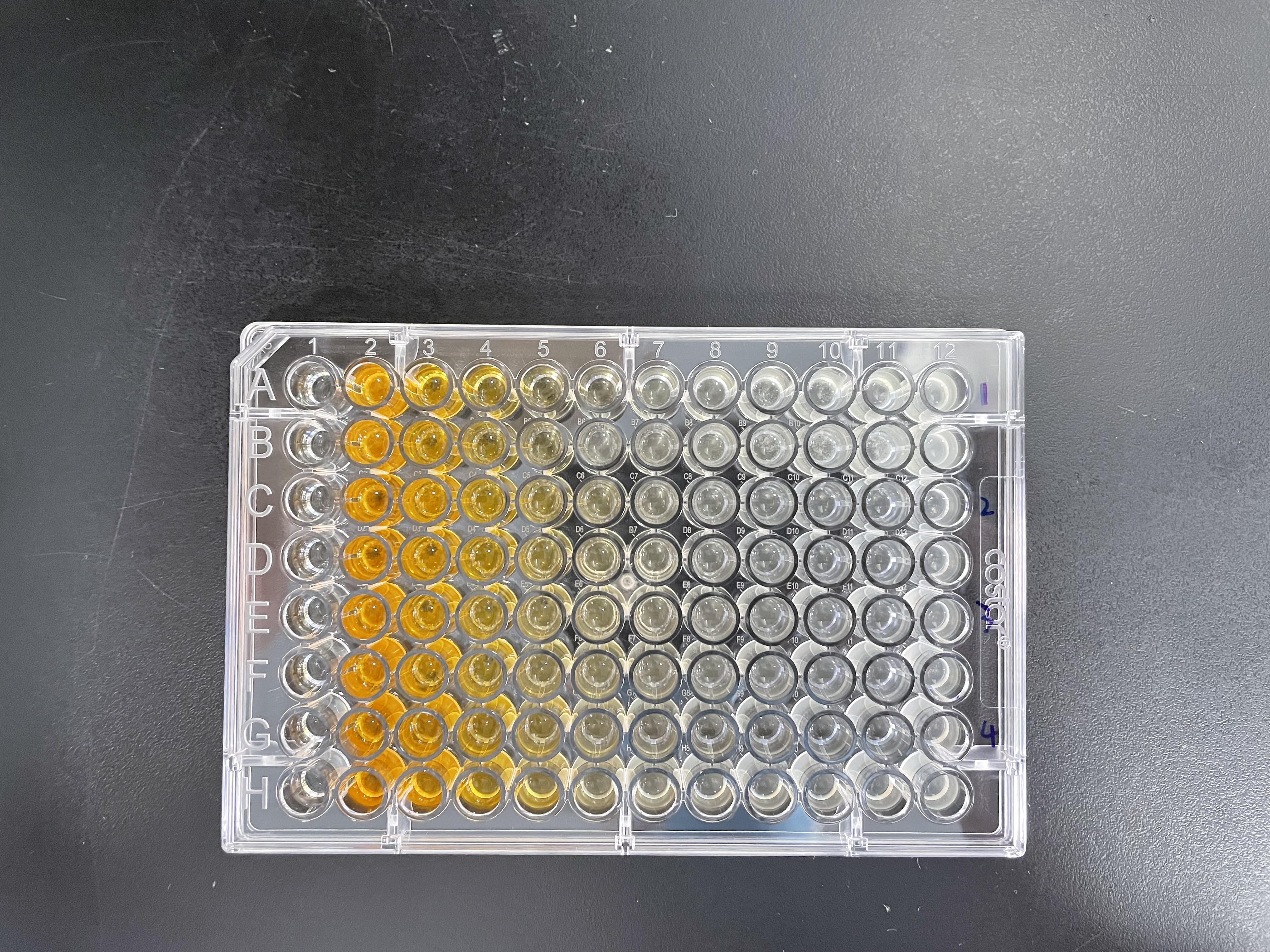

Supplement: Supplementary file 1 [file Data_Sheet_1.ZIP › Frontiers_raw data for Figure 1 and Table1-Gu Yeqing/Figure 1/MIC and MBC/MIC images.docx]

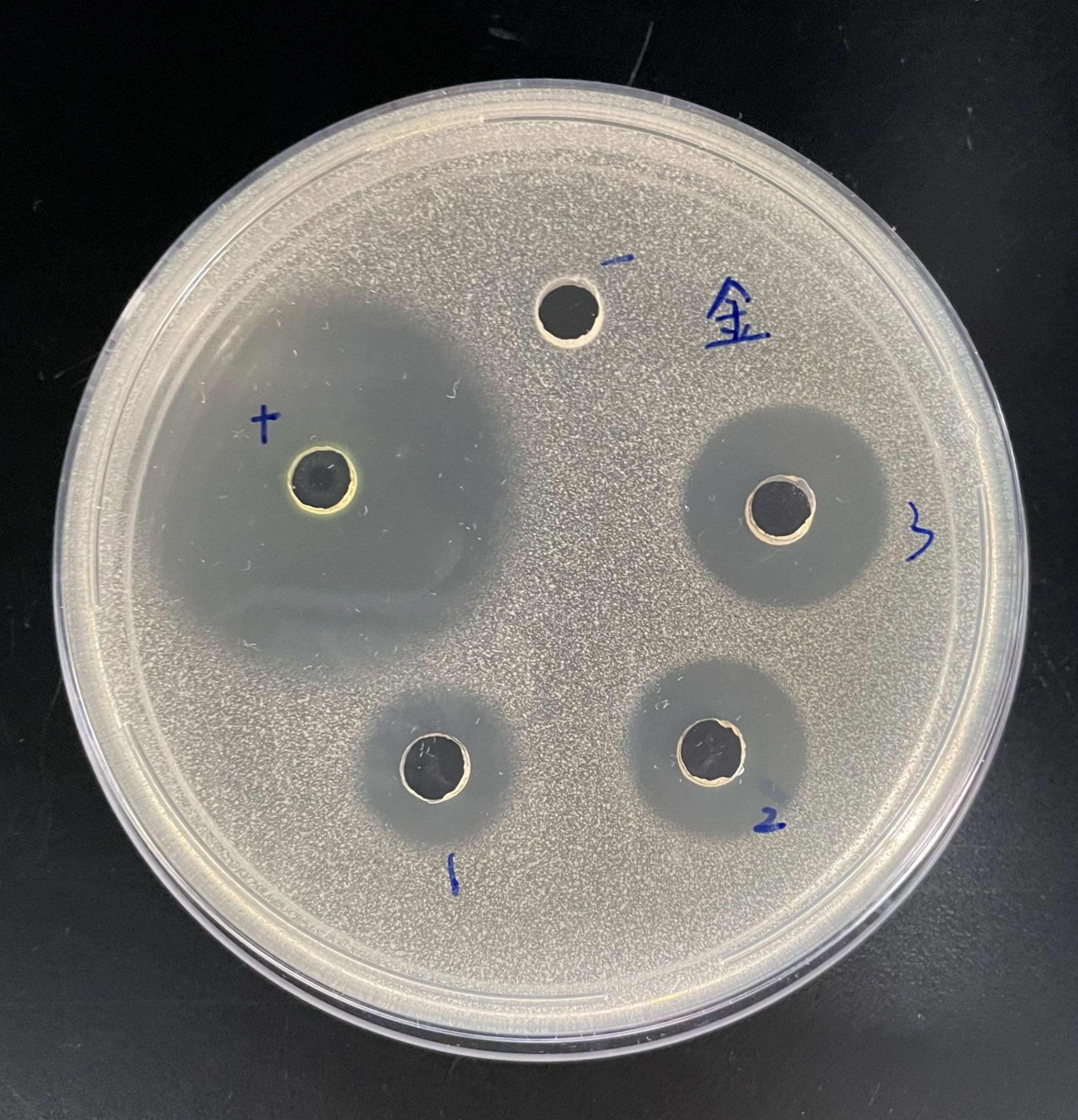

Supplement: Supplementary file 1 [file Data_Sheet_1.ZIP › Frontiers_raw data for Figure 1 and Table1-Gu Yeqing/Table 1/Inhibitory Zone Image 1.jpg]

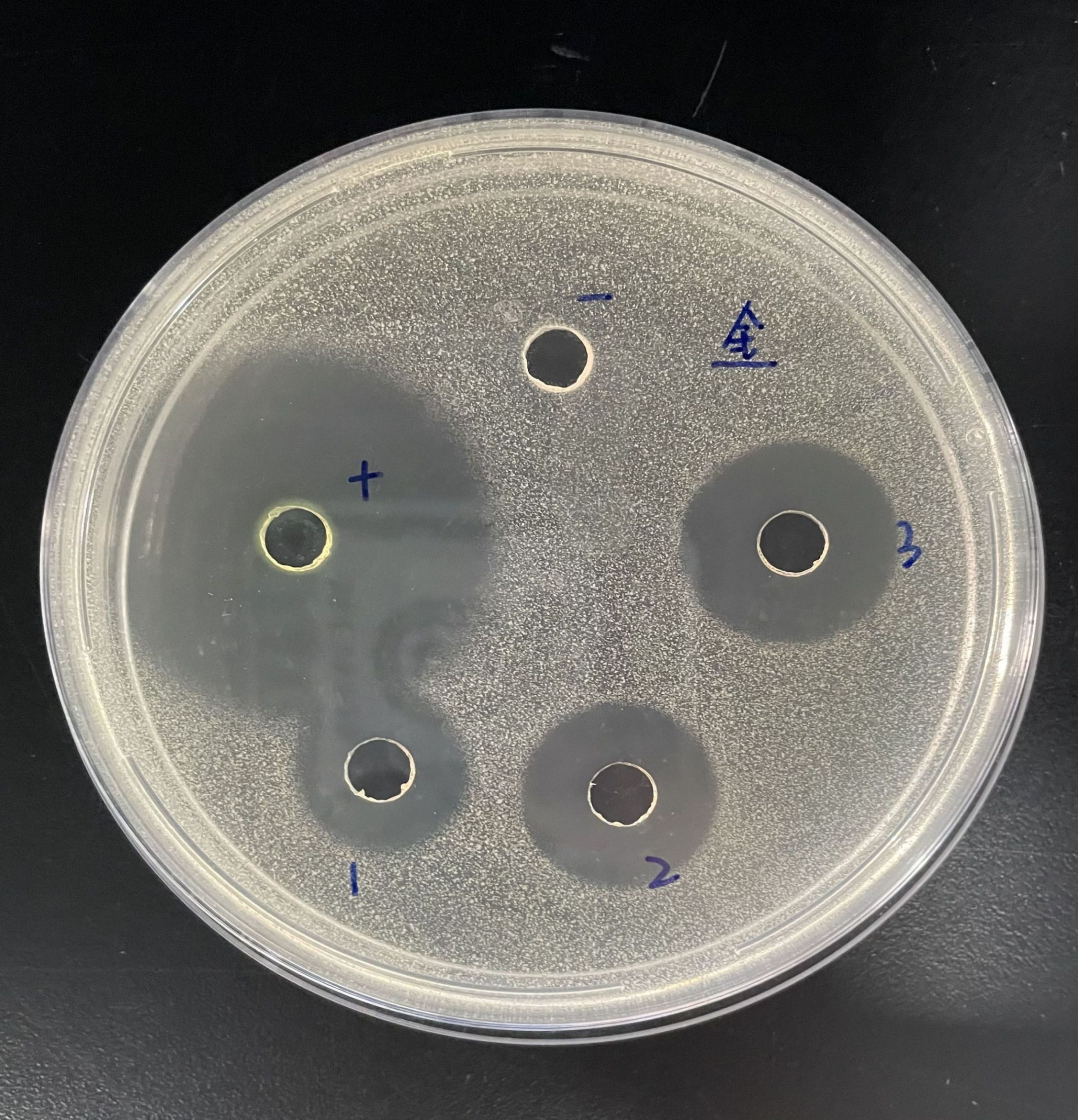

Supplement: Supplementary file 1 [file Data_Sheet_1.ZIP › Frontiers_raw data for Figure 1 and Table1-Gu Yeqing/Table 1/Inhibitory Zone Image 2.jpg]

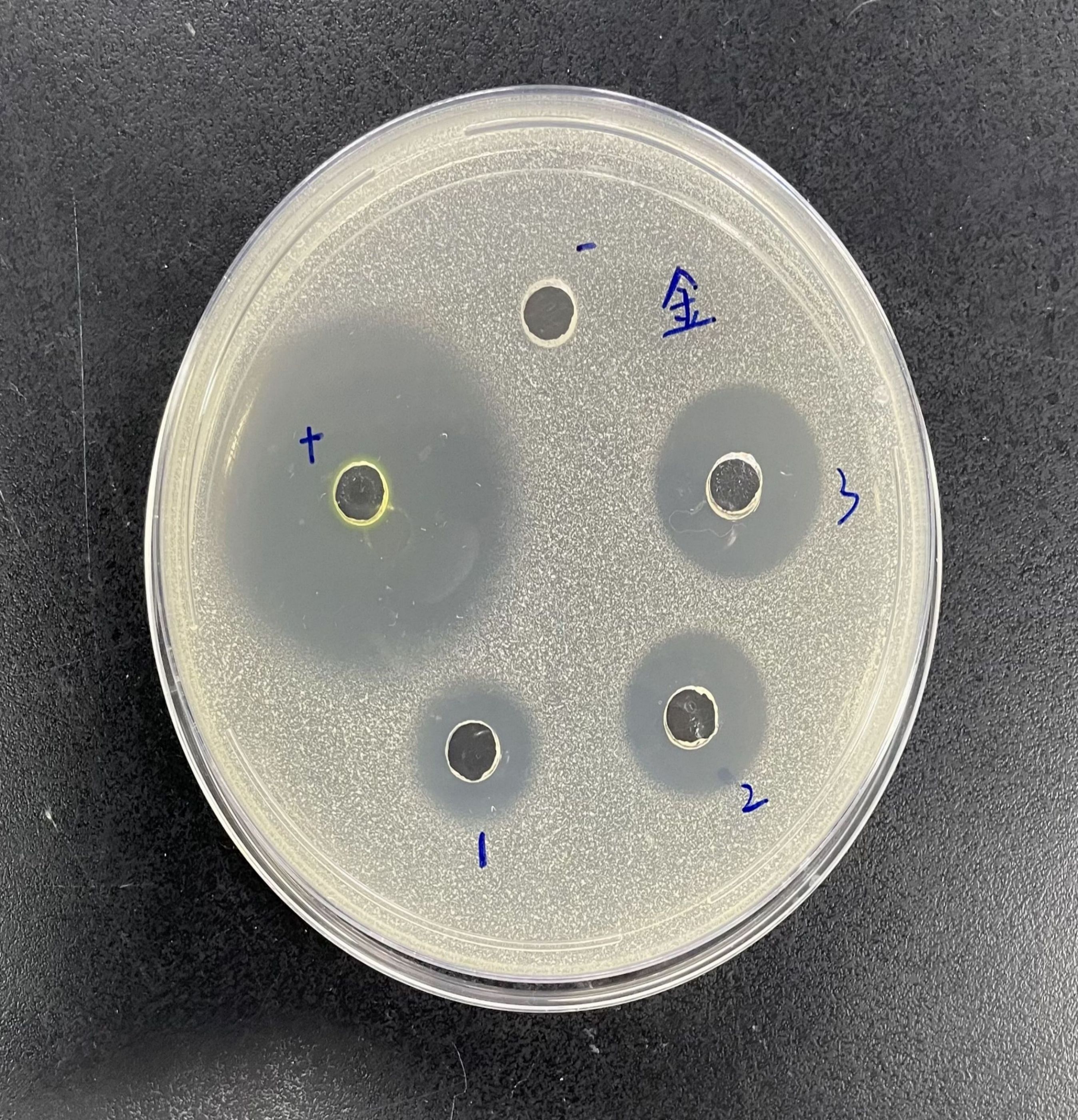

Supplement: Supplementary file 1 [file Data_Sheet_1.ZIP › Frontiers_raw data for Figure 1 and Table1-Gu Yeqing/Table 1/Inhibitory Zone Image 3.jpg]

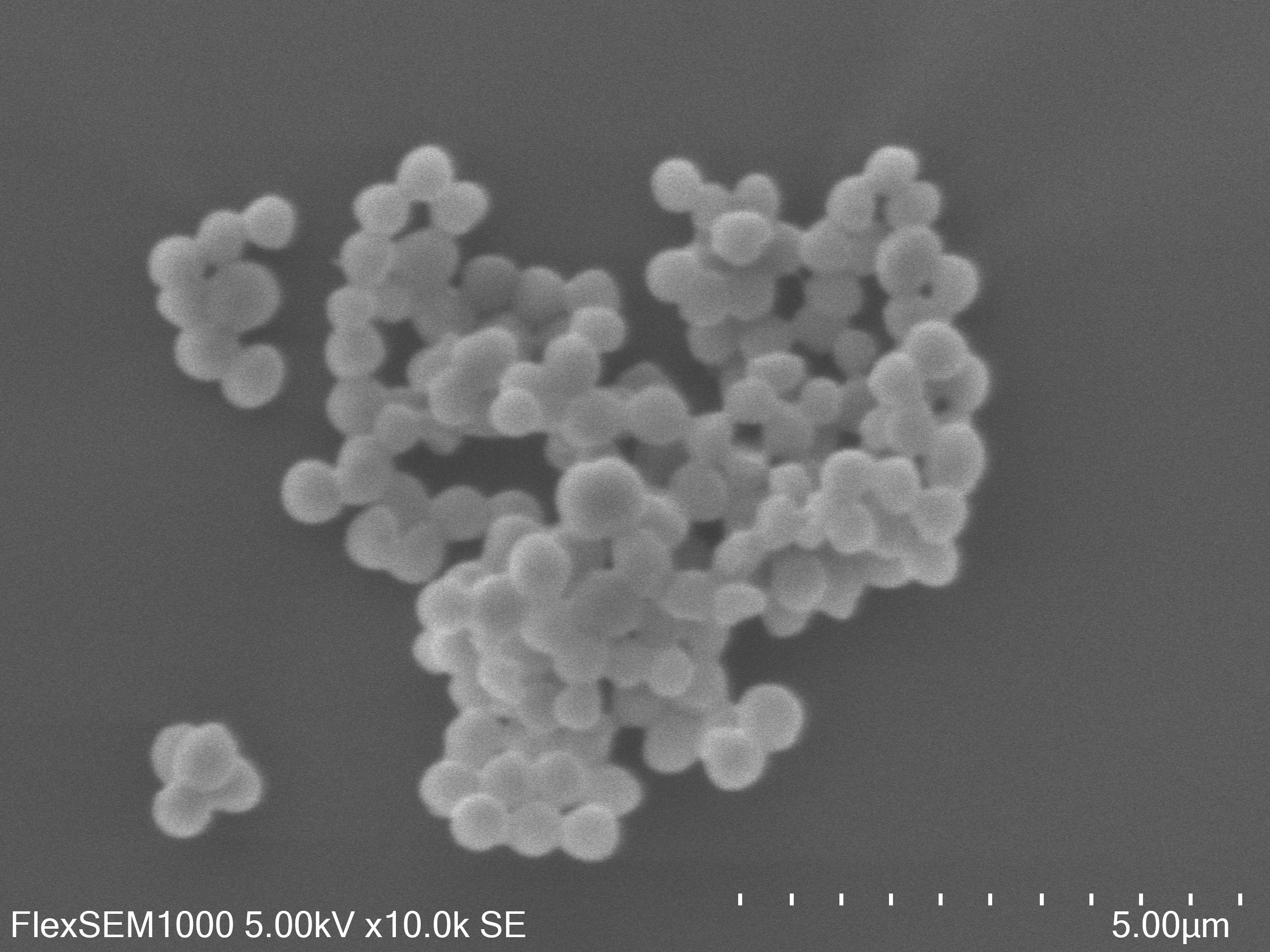

Supplement: Supplementary file 2 [file Data_Sheet_2.ZIP › Frontiers_raw data for Figure 2-5-Gu Yeqing/Figure 2/(A)0 × MIC.tif]

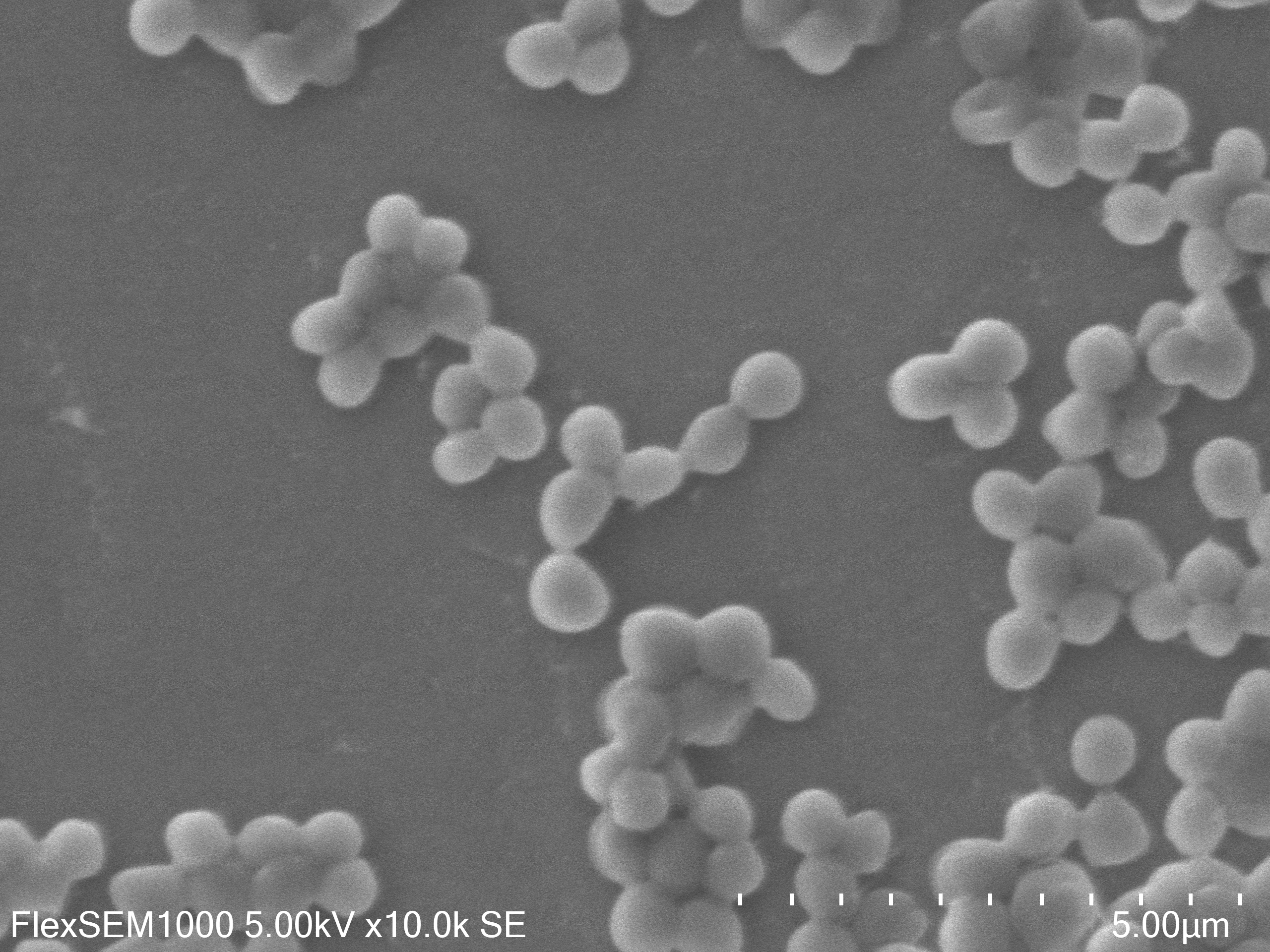

Supplement: Supplementary file 2 [file Data_Sheet_2.ZIP › Frontiers_raw data for Figure 2-5-Gu Yeqing/Figure 2/(B)2 × MIC.tif]

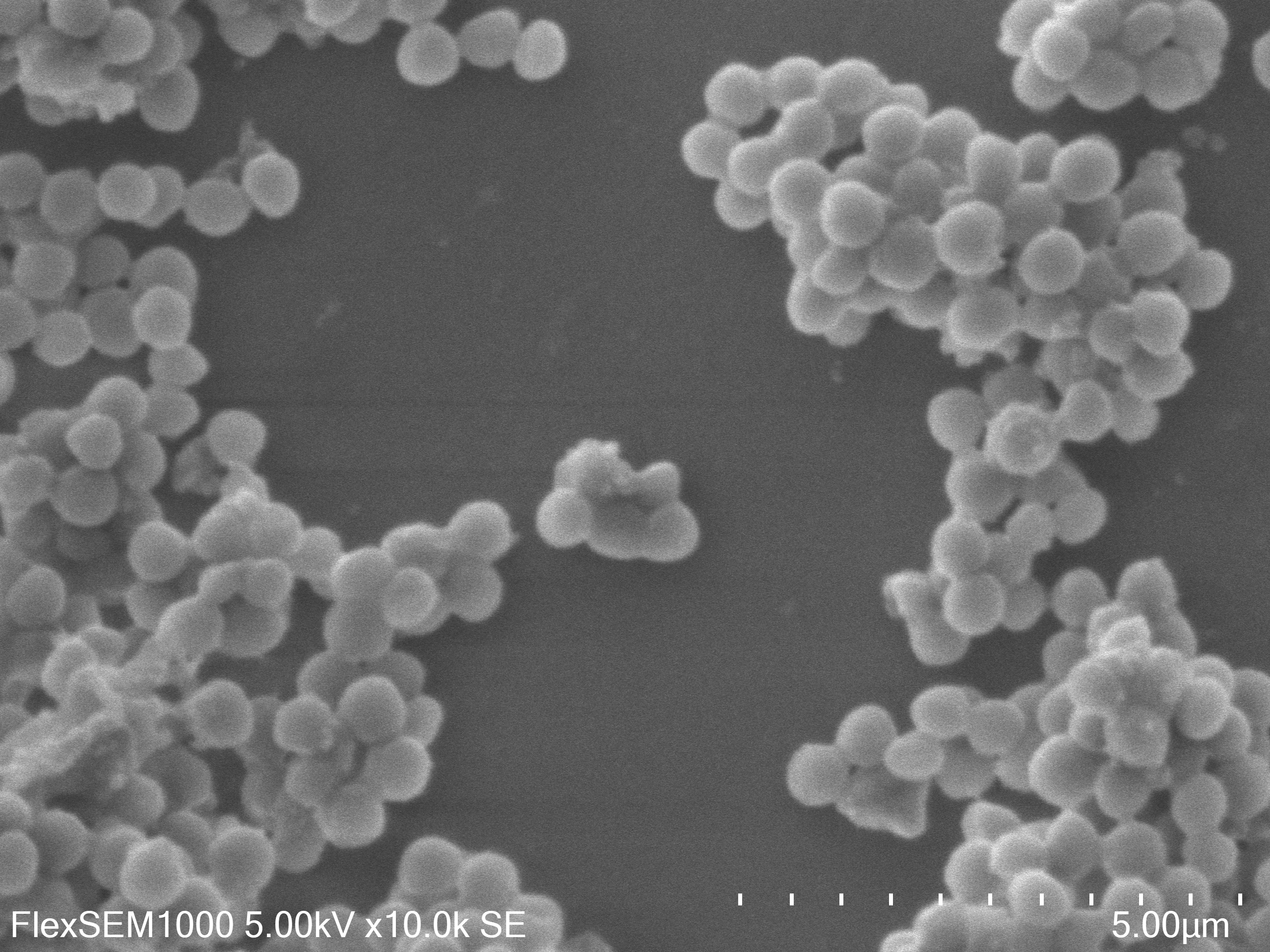

Supplement: Supplementary file 2 [file Data_Sheet_2.ZIP › Frontiers_raw data for Figure 2-5-Gu Yeqing/Figure 2/(C)4 × MIC.tif]

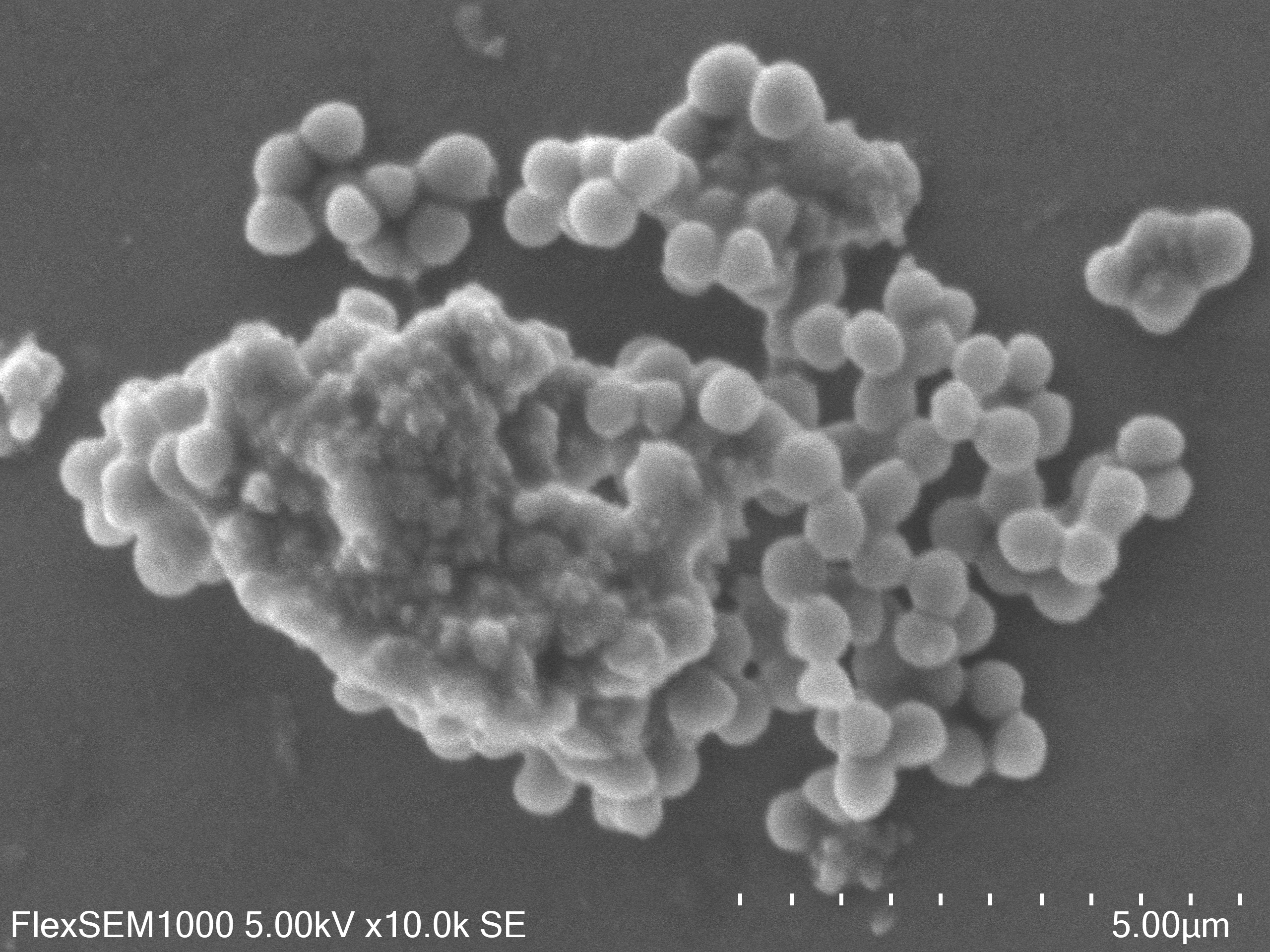

Supplement: Supplementary file 2 [file Data_Sheet_2.ZIP › Frontiers_raw data for Figure 2-5-Gu Yeqing/Figure 2/(D)8 × MIC.tif]

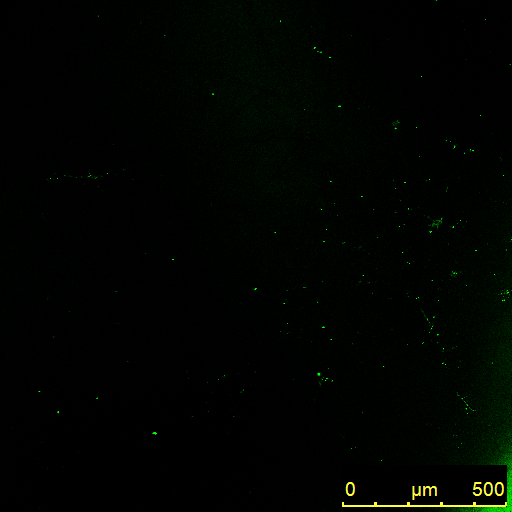

Supplement: Supplementary file 2 [file Data_Sheet_2.ZIP › Frontiers_raw data for Figure 2-5-Gu Yeqing/Figure 5/0 × MIC dark.tif]

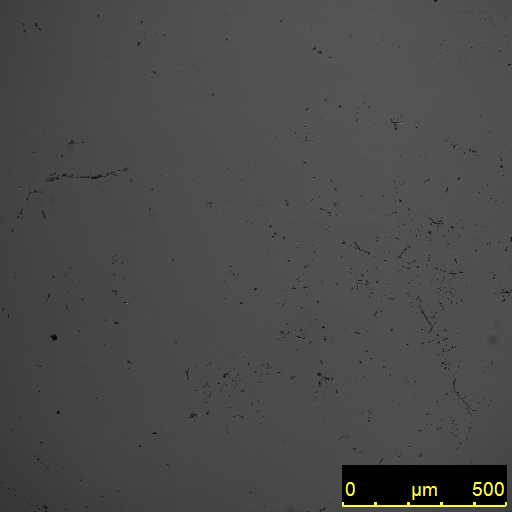

Supplement: Supplementary file 2 [file Data_Sheet_2.ZIP › Frontiers_raw data for Figure 2-5-Gu Yeqing/Figure 5/0 × MIC light.tif]

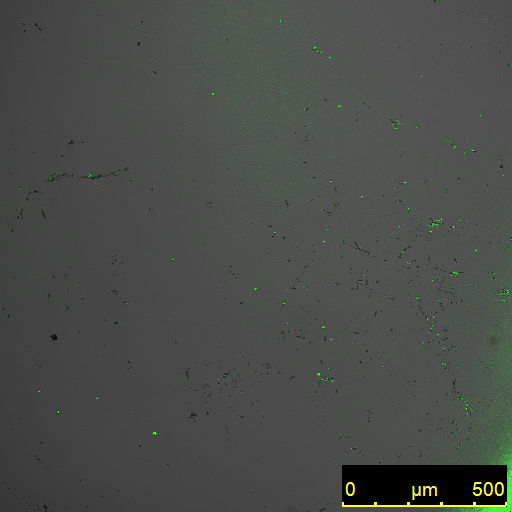

Supplement: Supplementary file 2 [file Data_Sheet_2.ZIP › Frontiers_raw data for Figure 2-5-Gu Yeqing/Figure 5/0 × MIC merged.tif]

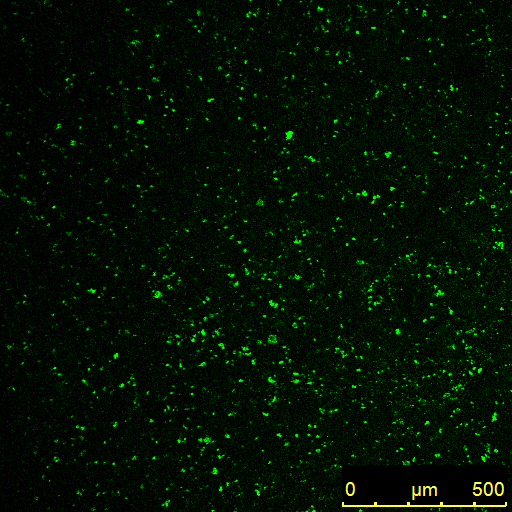

Supplement: Supplementary file 2 [file Data_Sheet_2.ZIP › Frontiers_raw data for Figure 2-5-Gu Yeqing/Figure 5/8 × MIC dark.tif]

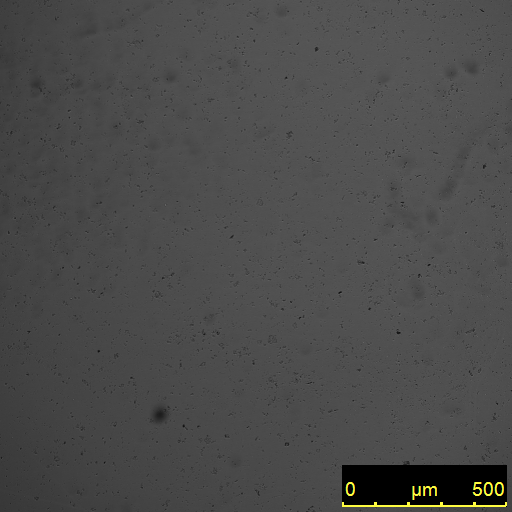

Supplement: Supplementary file 2 [file Data_Sheet_2.ZIP › Frontiers_raw data for Figure 2-5-Gu Yeqing/Figure 5/8 × MIC light.tif]

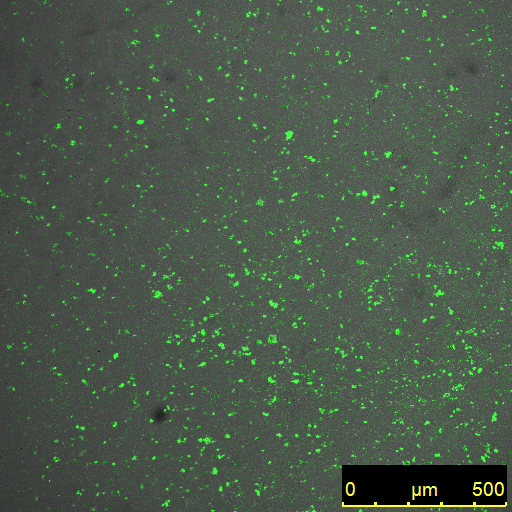

Supplement: Supplementary file 2 [file Data_Sheet_2.ZIP › Frontiers_raw data for Figure 2-5-Gu Yeqing/Figure 5/8 × MIC merged.tif]
